# The Relationship between Selected Parameters and the Occurrence of Premyopia in a Group of 1155 Children Aged 8 in Northwestern Poland

**DOI:** 10.3390/jcm13071977

**Published:** 2024-03-29

**Authors:** Monika Modrzejewska, Magdalena Durajczyk

**Affiliations:** Second Chair and Department of Ophthalmology, Pomeranian Medical University in Szczecin in Poland, 70-204 Szczecin, Poland; magdalena.durajczyk@gmail.com

**Keywords:** pre-myopia, accommodation, binocular vision, heterophoria, refractive errors

## Abstract

**Background:** Determination of the number of pupils at risk of developing pre-myopia and selected ophthalmic parameters in a group of 1155 children aged 8. **Material:** Ophthalmic examinations were performed in Polish 8-year-old, /1518 individuals/; 1155 of whom presented complete data for analysis. There was a total of 554 (47.9%) girls and 602 (52.1%) boys. Examination of the anterior and posterior segment of the eye, evaluation of accommodation, convergence, heterophoria, alignment of the eyeball, muscular balance with ocular mobility in 9 directions of gaze, and spatial vision were tested. Refraction was obtained under cycloplegia. Refractions (spherical equivalent, SE). were categorized as pre-myopia (−0.50 D–+0.75 D), myopia (≤−0.5 D), emmetropia (>−0.5 D to ≤+0.5 D), mildly hyperopia (>+0.5 D to ≤+2.0 D) and hyperopia (>+2.0 D). Data analysis was performed using Statistica 13.5 software: chi-squared, Pearson’s, *t*-Student, and U Mann–Whitney tests. *p*-values of <0.05 were considered statistically significant. **Results:** Pre-myopia was diagnosed in as many as 704 subjects (60.9%) with a similar frequency among both girls—328 (46.6%)—and boys with 376 (53.4%). **Conclusions:** Current data indicates that the growing group of myopic individuals in many industrialized countries is the sixth most common cause of blindness. Further research is crucial to understand the factors underlying accommodative and binocular mechanisms for myopia development and progression and to make recommendations for targeted interventions to slow the progression of myopia in a group of early school children.

## 1. Introduction

It is well-known that both genetic and environmental factors contribute to the development of myopia [1,2]. Currently, there are several factors that are not yet fully proven as causal in the development of myopia in children. These are short time spent outdoors [2,3,4,5,6,7], reduced outdoor physical activity, poor light exposure [5], reduced vitamin D and dopamine levels or UV radiation [1,8]. Genetic factors can also influence the development of myopia, as confirmed by Tkatchenko et al. [3], indicating that children with a myopic version of the APPP2 gene are five times more likely to develop myopia, while Bullimore M.A. et al. point to the heterogeneity of myopia with 70% of the variance in myopic refraction [9,10,11].

In addition to the aforementioned, the role of accommodation as a causal factor in the development and progression of myopia, resulting in excessive accommodative effort during prolonged or excessive near work, appears to be significant [12,13,14]. This aspect of myopia assessment indicates that insufficiently relaxed accommodation results in the focusing of distant images directly in front of the retina. The lack of proper correction of the myopic defect results in disruption of accommodation and/or delayed accommodation in myopia or an increase in the AC/A ratio. Accommodative gain and resulting lag can vary between children, with the possibility that myopic children accommodate less than emmetropic children [15]. Excessive tension of the ciliary muscle or the appearance of heterophoria in the absence of proper binocular vision results in blurred vision, which can cause asthenopic complaints: squinting, difficulty reading text, blurred vision of objects at a distance, changes in the curvature of the optical system of the eye, including myopization of the lens [14]. Other issues analyzed in the literature that are related to myopia and changes in accommodation are abnormal convergence and aspects of retinal blurring caused by delayed accommodation, the impact of close range, and insufficient working distance for myopia [16].

A known aspect at the intersection of the issues of myopia and accommodation is the state of binocular vision during work at near distances, which also varies depending on the amount of accommodation, though the effect of heterophoria during near work on the progression of myopia is not fully understood [17,18]. Esotropia, especially of the accommodative type, is related to hyperopia [19]. The relationship between myopia and exotropia was first proposed by Donders in 1899 [20] and further confirmed in more recent research findings [21].

Worldwide epidemiological data indicate that children and young people remain at an increased risk of developing myopia [14,22]. Data from Singapore indicate the incidence of myopia at 73.9% in a group of subjects aged 15–19 years [22]. Data from Poland indicate that in a group of 3970 schoolchildren from 7–15.5 years of age, the occurrence of myopia is reported at a level of 16.8% to 25.7% of the examined children [23]. According to the authors Modrzejewka and Durajczyk [24], a group of Polish 8-year-old children presented with myopia at an incidence of 16.8% and emmetropia at 39.7%. Norway data from 2019 shows an incidence of 32% for emmetropia and 17% for myopia in a group of patients 7–15 years. A study made in Bosnia and Herzegovina from 2017 shows myopia at 20.4% (7–16 years old). Ireland (2018–2019)—myopia at 19.9%. Sweden (2000)—myopia at 39% in two groups of subjects 6–7 and 12–13 years.

Hence, the search for factors that exacerbate myopia and prevent the formation of negative refraction detected in pre-myopia both before and after cycloplegia remains an important issue. The authors of this article performed a study of various parameters of the eye, such as determining the prevalence of refractive defects distinguishing pre-myopia, the presence of foria, analysis of changes in tests of binocular vision, deviation in the horizontal axis, changes in the refraction of the eye before and after the release of eye accommodation and also the relationship of selected parameters with the spherical equivalent in a group of 1518 children in early childhood.

## 2. Materials and Methods

In northwestern Poland, in Szczecin, ophthalmic screening was conducted, and 1518 8-year-old schoolchildren participated. We estimated the prevalence of refractive defects, selected parameters of the accommodating system of the eye, binocular vision, alignment of the eyeballs, and near and distance heterophoria. The research project was implemented in the 2017–2019 period and received a positive opinion from the Bioethics Committee of the Pomeranian Medical University in Szczecin (KB.006.25.2023, 1 March 2023). It was conducted in accordance with the tenets of the Declaration of Helsinki after gaining consent from the legal guardians of the children undergoing screening. Before the study began, the child’s caregiver was asked to fill out a questionnaire that asked about, among other things, ophthalmic diseases, past treatments, and eye conditions in the patient’s family. The ophthalmologic examination included an assessment of the following ophthalmologic parameters: visual acuity at near and distance (Snellen charts), two-fold examination of refractive defects using an auto-refractometer (Retinomax 3rd, SN 2202005 Tokyo, Japan 2012) before and after the use of cycloplegic drops (Tropicamide 1% at 1 drop 3 times every 5 min according to the standards of accepted refractive tests, tropicamide can be considered a viable substitute for cyclopentolate [25,26]), evaluation of the anterior segment of the eye in a hand-held slit lamp, evaluation of the fundus (direct and indirect ophthalmoscopy), evaluation of accommodation (examination of the near point of each eye—three-fold repetitions), three-fold examination of convergent accommodation, evaluation of eye alignment and muscular balance (unilateral and alternating cover-test), examination of ocular mobility in 9 directions of gaze, examination in the heteroforia by means of the Maddox test for a distance of 5 m and the Maddox test for a distance of 30 cm. A Titmus Test was performed to assess spatial vision.

Statistical analysis of the data was performed using the chi-squared test, the Student’s *t*-test, and the Kruskal–Wallis test in Statistics 13.5. Values of *p* < 0.05 were considered statistically significant.

For the above study, the criteria of Spherical Equivalent of Cylindrical Power (SNO, calculated as the sum of half the cylindrical power and spherical power expressed in spherical diopters—Dsph) after total cycloplegia were used to assess refractive defects in children. Based on the studies of IMI [27], the criteria for diagnosing a refractive defect were adopted.

## 3. Results

**Figure 1:**
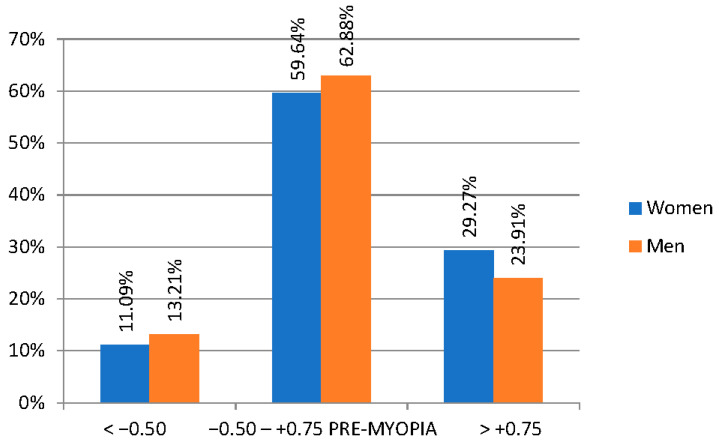
Prevalence of pre−myopia in the study population by gender. Not statistically significant between groups. *p* = 0.09759.

The study included 1518 elementary school second-graders (*n* = 3036 eyes), 1155 of whom presented complete data for analysis. There was a total of 554 (47.9%) girls and 602 (52.1%) boys. The average age of children participating in the study was 8.1 years (SD = 0.44). All examined children were Caucasian from urban and suburban areas. Following the aforementioned criteria for classifying refractive defects in children (Table 1), the following results were obtained for the prevalence of visual defects in the study population (post-cycloplegic auto-refractometer): mild hyperopia—398 children (34.4%), myopia—140 children (12.1%), significant hyperopia—56 children (4.8%). Emmetropia occurred among 554 children (47.9%). Pre-myopia was diagnosed in as many as 704 subjects (60.9%) with a similar frequency in girls 328 and boys 376—Table 2, Figure 1.

**Figure 2:**
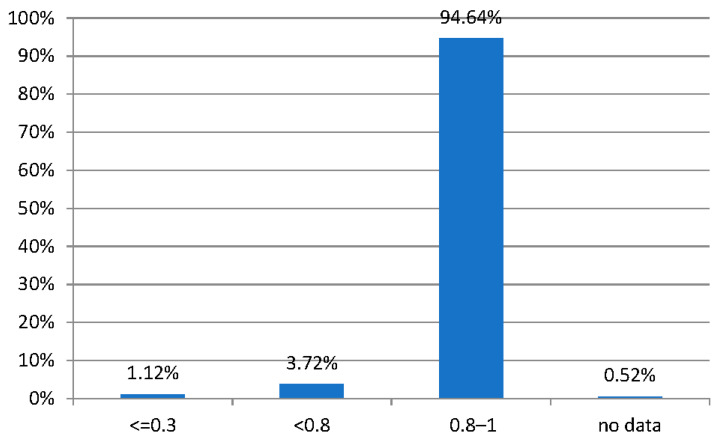
Breakdown based on distance visual acuity with Snellen chart.

Of those examined, 1094 (94.6%) had visual acuity with or without the use of a correction of 0.8–1.0 (according to Snellen), 43 children (3.7%) had visual acuity <0.8–0.3, and 13 (1.12%) had ≤0.3 (Figure 2).

There was a statistically significant relationship between visual acuity for distance and wearing glasses (*p* < 0.001). In the group without glasses, almost all subjects had visual acuity in the range of 0.80–1.00 (96.28% of the group without glasses), while 2.79% had acuity above 0.3 and below 0.8, and 0.93% of the group without glasses had visual acuity not exceeding to 0.30. In contrast, 78.67% of subjects with visual acuity at a level of 0.80–1.00 (Snellen tables) were recorded in the group of subjects with selected eyeglass correction. At the same time, in this group, 17.33% of subjects with visual acuity in the range between 0.30 and 0.8 (Snellen tables) were recorded wearing glasses, while 4% of subjects wearing glasses had a visual acuity to distance at <0.30.

Constant strabismus occurred in 18 children (1.55%), and intermittent strabismus was present among 66 students (5.7%), with a similar frequency in girls and boys. Statistically, significantly more often (*p* = 0.00000), constant strabismus was detected in children using ocular correction. In addition, there was a statistically significant relationship between the positive alternate cover test and the spherical equivalent (*p* = 0.036).

**Figure 3:**
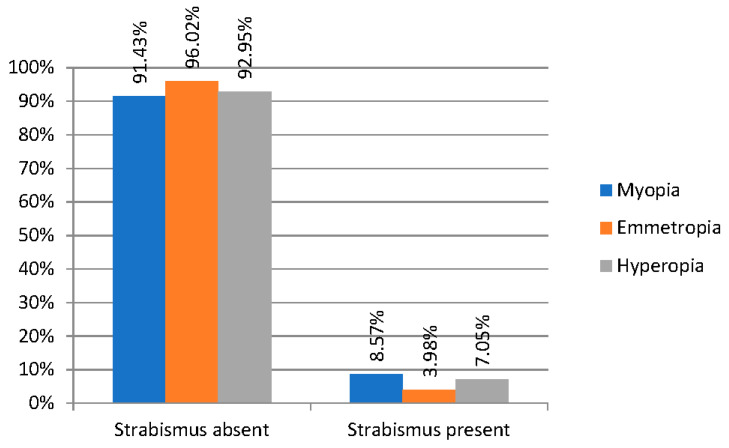
Prevalence of strabismus in different refractive errors.

In the three groups of refractive errors under comparison (myopic, emmetropic, and hyperopic), horizontal strabismus occurs with varying frequency. In our results, it can be observed that in the group of children with emmetropia, horizontal strabismus occurs less frequently (in 3.98%) than in the myopic group (8.57% of the subjects) and hyperopic patients (7.05%) (Figure 3).

Eye deviation > 0° tested with the Maddox test to the near distance could be observed in 496 subjects (42.9%), of which deviation > 5° was observed in 15 children (1.29%) with similar frequency in girls and boys (*p* = 0.89869). Binocular vision tested with the Titmus test was impaired in 9 subjects (0.77%) of the study population. There was a statistically significantly higher frequency (*p* = 0.037) of the Maddox score > 5° in the group with a negative Titmus test than in the group with a positive Titmus test.

In each of the four groups, those of emmetropia, mild hyperopia, significant hyperopia, and myopia, there was a statistically significant difference between the result of the spherical equivalent before the drops were administered and that of the spherical equivalent after administration (*p* < 0.001). Based on descriptive statistics, it can be observed that there was a statistically significant increase in the equivalent value in all groups.

Myopia is more likely to occur in children who showed “instrumental myopia”, which means at least −1.00 Dsph when tested with an auto-refractometer. Due to the difficulty of determining the moment when myopia begins to appear, it is hard to analyze test results preceding the appearance of this defect.

In our results, instrumental myopia (a score of less than −1.00 Dsph) was observed in 99 children (8.56%). By contrast, in 38 children (3.28%), there was no difference in the equivalent before and after administration of the drops.

At the limit of statistical significance (*p* = 0.0509), a correlation was observed that in the group with instrument myopia, there are slightly more subjects with cases of myopia in the family (as many as 30.3%) compared to the group without instrument myopia (21.73%).

There was no statistically significant relationship between the presence of strabismus (positive Cover Test, Maddox, Tytmus test) and instrument myopia (*p* = 0.372).

**Figure 4:**
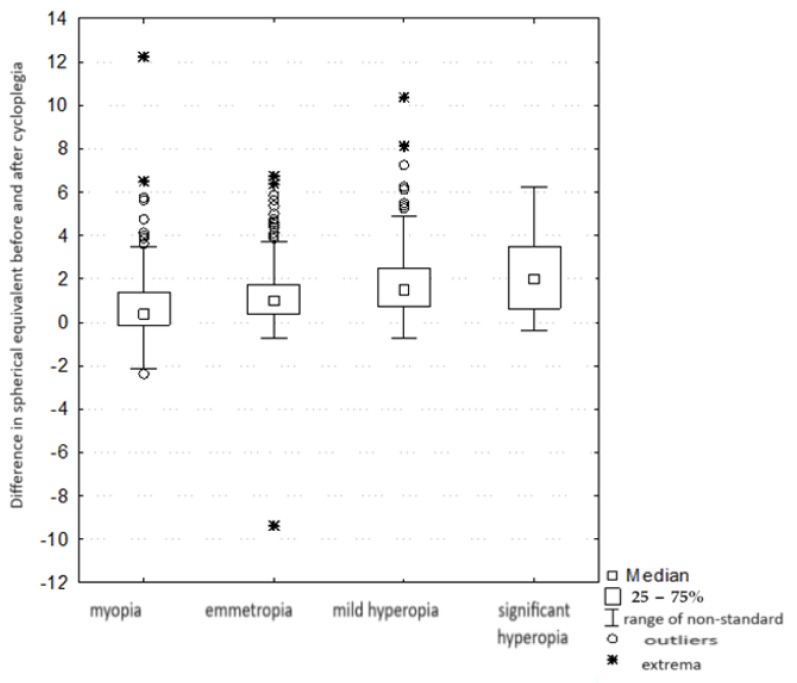
Comparison of the magnitude of the change in equivalents between groups with different refractive defects.

There was a statistically significant difference (H = 93.403, *p* < 0.001) (Table 3) in the “magnitude of the equivalent change” between the groups with different refractive defects—that is, the difference between the equivalent refractive values before and after cycloplegia. Differences were noted between the myopic and emmetropic groups (with the amount of change in the myopia group being smaller than in the emmetropia group). There was no statistically significant difference in the magnitude of the change in equivalents between the groups with mild and significant hyperopia. There were also differences between groups, between IV and II (here also IV < II), and IV and III (here also IV < III). In addition, there was also a difference between groups I and II (I < II) and I and III (I < III). There was no statistically significant difference in the magnitude of the change in equivalents between groups II and III (that is, in group II, the magnitude of the change between pre- and post-drop equivalents was similar to the magnitude of the change between these equivalents in group III). The data is shown in Figure 4.

**Figure 5:**
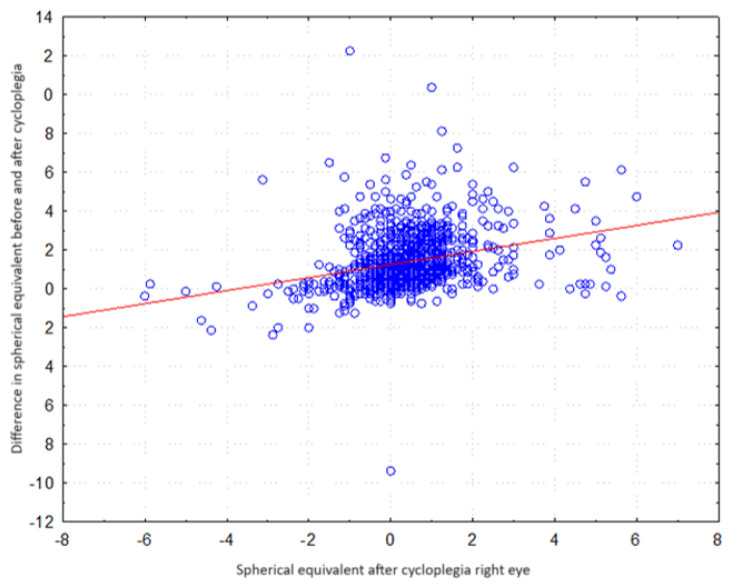
Scatter plot. Correlation of the difference in mean spherical equivalents between hyperopic, myopic, and emmetropic subjects.

There was a statistically significant correlation between the difference in spherical equivalent values before and after cycloplegia (R = 0.313, *p* < 0.001). Higher differences in equivalents (post-cycloplegia) corresponded to higher equivalent scores after cycloplegia. The data is shown in Figure 5.

## 4. Discussion

The results of a number of studies indicate that one of the mechanisms responsible for myopia of the eye remains a disturbed process of emmetropization to myopia, altering the power of the optic system, tensing the ciliary muscle via the choroidal-center response, which is one of the drivers of eyeball elongation [28] in addition to mechanical stretching of the eyeball stimulated by an increase in intraocular pressure with excessive accommodative tension during myopia [29], which has not been fully confirmed in the conducted studies [30].

The generation of strong accommodative contraction during intense close-up work can result in myopia in the hyperopic or emmetropic eye [31], as well as increased myopia in the hitherto myopic eye. Children and young adults, due to their high amplitude of accommodation coefficient, are particularly prone to pseudomyopia (which is the result of an increase in the refractive power of the eye due to excessive stimulation of the accommodation mechanism), which, according to the literature, can occur with varying frequency [24,32,33].

The results of the research by the authors Modrzejewska and Durajczyk on refraction of 8-year-old early school children indicate that the incidence of pseudomyopia was 51.91% [24], and the mean SE before and after the cycloplegic drops was −1.78 D and −0.8 D, respectively, indicating that up to 68.71% of the early school-age pediatric population may develop myopia in the future. An important element in the above study is the indication that as many as 60.9% of the subjects are in the “pre-myopia” range, so these are young people who can probably be protected from the onset of myopia, which, according to the IMI remains a primary goal for today [34]. Currently, researchers are focusing on inhibiting the progression of pre-myopia using 0.01% atropine [35]. Already in the early 1960s, Hirsch [36] noted in his study that children with low hyperopia became more often myopic than others during their school years. Goss also conducted a search to predict the development of myopia [37]. He conducted a study on 75 subjects who became myopic over time and 75 subjects who were already myopic at the same time. He observed a statistically significant difference in the results of the two groups for heterophoria to myopia. Those who became myopic showed a mean value of 1.0 prismatic diopters dissociated esophoria to near, while those who remained emmetropic obtained a mean value of this heterophoria at 2.0 prismatic diopters base to nose. Positive relative accommodation was found to be significantly lower in the group of subjects who became myopic compared to those who remained emmetropic. Research in predicting the development of myopia was also undertaken by Drobe and Saint-Andre [38]. They studied a group of 25 people who became myopic and compared the results to a group of 25 people who remained myopic. They observed that myopic individuals had less and decreasing hyperopia, problems with accommodation, and lower exophoria values with a tendency toward esophoria before the defect appeared. Similarly, Zadnik et al. showed that children who have low hyperopia when starting school are more likely to develop myopia than others [39].

A common refraction test with an auto-refractometer shows a high frequency of erroneous test results, which is the result of excessive psychological accommodation observed in a group of preschool and early school children, which, in turn, is inhibited by the paralysis of eye accommodation. In the work reported here, the auto-refractometer test was performed before and after the paralysis of accommodation to note what share of the negative refraction resulted from excessive accommodation. This is the so-called share of instrumental accommodation, which is partly responsible for the appearance of negative refraction of the eye as a result of the summation of the action of both types of accommodation. These are key parameters before the appearance of myopia. Psychological instrumental accommodation is defined as the component of accommodation initiated by judging the distance at which the observed object is located [40]. Studies by Rosenfield and Gilmartin [41], Wick and Currie [42], and Rosenfield and Ciuffreda [43] have confirmed the effect of psychological accommodation on the increase in accommodative responses. One of the few studies on the effect of psychological accommodation and its influence on the development of myopia in people working up to myopia with a microscope is that of Ting et al. [44]. The same happens when looking through a telescope, phoropter, and an auto-refractometer. These authors found no statistically significant correlations between instrumental myopia and convergence, myopia, foria, AC/A fraction (the ratio of accommodative convergence to stimulus to accommodation), inadequate accommodation, or tonic accommodation. Significantly, the likelihood of myopia is higher in children who showed instrumental myopia greater than −1.00 dptr when tested with an auto-refractometer [45]. In our study, as many as 8.56% of children showed instrumental myopia, but as in the studies cited above, no statistically significant correlations were found between instrumental myopia and convergence, near-field foria, or binocular vision.

Having binocular vision is extremely important in producing a correct retinal image. Binocularity improves the accommodative response to defocus, and in turn, blur caused by defocus is a useful cue in the absence of binocular vision. This effect may be different in people with myopia [46]. For example, sensitivity to blur is reduced in myopia under monocular but not binocular conditions [47]. According to Vera-Diaz F.A. et al., myopia also shows reduced stereopsis with flickering stimuli and greater binocular imbalance compared to emmetropes [48]. This relationship was not demonstrated in our study.

Theoretically, a higher AC/A value may also cause the eyes to shift toward esophoria when working at close range in myopic children. Fusiform vergence is also higher in those with progressive myopia [49], and on the other hand, myopic children show less convergent changes in vergence adaptation compared to emmetropes, which may be attributed to higher accommodative adaptation (assessed by changes in tonic accommodation) [50]. When myopia is compensated with spectacle correction for distance, a child’s area of clear single binocular vision becomes more divergent, and accommodative responses increase relative to that measured with correction with mono-focal glasses [51]. A limitation of our work is that examiners enter absolute values of eyeball deviation without marking the direction of exo- or esophoria.

Relative peripheral refraction, measured as the difference between foveal and peripheral refractive error, is known to have a significant effect on the development and control of myopia. Myopic individuals tend to have the relative peripheral refraction of hyperopia, while hyperopic individuals have the relative peripheral refraction of myopia [14]. Eye shape with accommodation and accommodation delay can further affect peripheral refractive error, and changes in aberration with accommodation can also affect off-axis refractive errors during accommodation. Myopic individuals are likely to have a larger ciliary muscle mass, so accommodation may lead to an expansion of the myopic eye’s dimensions due to the force produced by the larger ciliary muscle. This would lead to changes in relative peripheral refraction in people with myopia. As discussed earlier, the evidence for clinically significant changes in axial length and central refractive error during myopia is inconclusive. The discrepancies in these studies can be attributed to the level of myopia with significant differences in highly myopic eyes [52]. The sensitivity to defocus in the peripheral retina may be lower than that of the central retina due to fewer cones and ganglion cells. The quality of vision decreases with the angle of the visual field, so the resolution of peripheral vision is low and has less sensitivity to defocus [46]. Depth of field in the peripheral field up to 45 degrees remains at ±1.0 D, which explains that any change in peripheral focus above ±1.0 D is likely to be perceived as defocused in the peripheral retina and may interfere with emmetropization [53].

Binocular vision is an important tool in retinal image formation, improving the accommodative response to defocus [54], and, therefore, probably has an impact on emmetropization and myopia [55]. Proper binocular vision contributes significantly to postural stability control and proper motor balance via postural control and proper motor coordination [56]. Individuals with myopia show greater postural instability to peripheral stimuli and distortions presented in the stimuli than those with emmetropia, as confirmed in a study by Modrzejewska M. et al. [56]. In our results, binocular vision impairment was present in 0.77% of the study population.

All modern optical interventions used for myopia are based on the common assumption that reducing off-axis hyperopia blur or inducing off-axis myopia blur should slow the progression of myopia [57]. Optical designs incorporate one or more paracentral or peripheral zones around a central bright zone to induce peripheral areas or simultaneous myopic blur in the retina while providing clear on-axis focus and off-center vision. Such dual-powered designs have the potential to disrupt the accommodative and binaural systems, as children with myopia may not accommodate when looking through relative positive zones, further impairing potentially reduced accommodative function due to myopia.

Several studies have evaluated the effect of soft bifocal or multifocal contact lenses on the accommodative response in adults, but the results are mixed. Some studies have shown either a similar response to wearing contact lenses for one visual field [58] or a predominance of accommodation [59]. Others have shown increased accommodative delay [60], decreased ease of monocular accommodation [61], and exophoric shifts at near [60].

## 5. Conclusions

Further research is crucial to understand the factors underlying accommodative and binocular mechanisms for myopia development and progression and to make recommendations for targeted interventions to slow the progression of myopia in a group of early school children.

Current data indicates that the growing group of myopic individuals in many industrialized countries is the sixth most common cause of blindness, associated mainly with complications correlating with axial elongation of the eyeball and degenerative changes in the retina due to deficits in ocular circulation. Thus, it is suggested that understanding the etiology, epidemiology, and results of various treatment regimens may modify current care and result in reduced morbidity due to progressive myopia. Current animal and human studies show that the development of myopia is the result of the interaction of genetic and environmental factors. The prevalence of myopia is higher in people whose both parents are myopic, suggesting that genetic factors are clearly involved in the development of myopia. At the same time, population-based studies suggest that the development of myopia is related to education and the amount of time spent near work. Thus, activities performed at close distances render one susceptible to an increase in the accommodation coefficient, a decrease in convergence ability, and exposure to optical blur.

## Figures and Tables

**Table 1:** Criteria for refractive errors, according to IMI [27].

Refraction Parameter	Spherical Equivalent (D)
Emmetropia	−0.5 to ≤+0.5°
Pre-myopia	−0.50 D–+0.75
Myopia	≥−0.50
Mild hyperopia	≥+0.50–≤+2.0
Significant hyperopia	≥+2.00

**Table 2:** Prevalence of pre-myopia in children in the study population by gender. Not statistically significant between groups *p* = 0.09759.

Spherical Equivalent after Drops, Right Eye	Gender (M/F) F	Gender (M/F) M	Overall
below −0.50	61	79	140
from −0.50 to +0.75 PRE-MYOPIA	328	376	704
above +0.75	161	143	304
overall	550	598	1148
Chi^2^ Pearsona	4.653970	df = 2	*p* = 0.09759

**Table 3:** Comparison of the magnitude of change in equivalents between groups with different refractive errors.

	Comparison of the Magnitude of the Change in Equivalents between Groups with Different Refractive Errors Kruskal–Wallis Test: H (3, *n* = 1144) = 93.40315 *p* = 0.000			
	Myopia R: 396.78	Emmetropia R: 531.25	Mild Hyperopia R: 670.19	Significant Hyperopia R: 717.90
Myopia (group IV)		0.000114	0.000000	0.000000
Emmetropia (group I)	0.000114		0.000000	0.000337
mild hyperopia (group II)	0.000000	0.000000		1.000000
significant hyperopia (group III)	0.000000	0.000337	1.000000	

## Data Availability

The data used to support the findings of this study are available from the corresponding author upon request.
